# Supplementary material for: Observation of second sound in graphite over 200 K
Source: Nat Commun. 2022 Jan 12;13:285. doi: 10.1038/s41467-021-27907-z (PMC8755757; doi:10.1038/s41467-021-27907-z)
Supplement: Supplementary file 1 — Supplementary Information [file 41467_2021_27907_MOESM1_ESM.pdf]

Supplementary Materials

Observation of Second Sound in Graphite over 200 K

Zhiwei Ding,[1†] Ke Chen,[1†] Bai Song,[1] Jungwoo Shin,[1] Alexei A. Maznev,[2] Keith A. Nelson,[2*] and Gang Chen[1*]

[1]Department of Mechanical Engineering
[2]Department of Chemistry
Massachusetts Institute of Technology
Cambridge, MA 02139

† These authors contributed equally to this work
* Corresponding author: kanelson@mit.edu; gchen2@mit.edu

1. Thermal transient grating measurements and analysis

The measured second sound signals using the upgraded TTG system are shown in Fig. S2a. By changing the heterodyne phase difference between the reference beam and the probe light, one could identify and separate the amplitude grating and phase grating signals in the TTG measurement, representing the change of the real and the imaginary part of the complex reflection coefficient, respectively[1]. As discussed in Ref. 2, because the thermoreflectance coefficient of graphite is quite small, the red curve in Fig. S2a, which shows only spikes at zero time delay and oscillations around 0 after that, should be the "amplitude grating" signal, and the black curve, which is taken at maximized second peak, should be the "phase grating" signal. The first peak of the black curve can be attributed to the excitation of electron-hole pairs, while the second peak of the black curve indicates the build-up of the temperature grating profile through electron-phonon and phonon-phonon scattering. The red curve shows obvious oscillations, corresponding to a speed of ~20600 m/s, very close to sound speed of the LA mode in graphite (~21000 m/s). One possible interpretation of this oscillation is that the in-plane standing wave modulates the density and thus the refractive index, and such modulation is sensed by the real part of the reflectance and contributes to the amplitude signal. Fast Fourier transformation analysis shows that the black curve also has the oscillation at this frequency but with a weaker magnitude, which may arise from a slight mixing of amplitude grating as the heterodyne phase might not be perfectly set at the pure phase grating point. In order to filter out this unwanted oscillation, we adopt the Savitzky-Golay method[3] to smooth our phase grating signal, as shown in Fig. S2b. It can be seen that the smoothed curve captures the profile of our raw data but filters out the high frequency acoustic oscillations. With the smoothed curve, we can clearly identify the position and the magnitude of the second sound "dip". All our measurements and data processing were performed as described in this section.

The statistical error of the data points in the signal waveforms can be estimated by the standard deviation of the negative-time-delay signals, as shown in Fig. S2b. In our pump-probe experiments, the negative-time-delay signals should be 0 in the ideal case. While in the real case, the randomness of the measurement system cause fluctuation around the 0 baseline (Fig. S2b). The magnitude of the fluctuation could be used to estimate the measurement errors. Most of our measurement errors are around 0.01, while the "dip" amplitude of those signals which we claimed to be second sound observations are much larger than their errors, indicating the "dips" are intrinsic and reliable.

2. Temperature response calculation

All the calculation are based on the temperature response with an arbitrary heating profile derived in Ref.4. Here we briefly recap its derivation.

We start with the PBTE,

$$\frac{\partial f_\mu}{\partial t} + \overrightarrow{\mathbf{v}_\mu} \cdot \nabla f_\mu = Q_\mu \frac{Nv}{\hbar\omega_\mu} + \sum_\nu W_{\mu\nu}(f_\nu^0 - f_\nu) \qquad \text{(S1)}$$

where μ is a short-hand index for a given phonon mode (branch and wavevector in the Brillouin zone), ω_μ is the frequency of the given phonon mode, f_μ is the non-equilibrium distribution function, N is the number of discretized points in the Brillouin zone, v is the unit cell volume and f_μ^0 is the equilibrium (Bose-Einstein) distribution function. **W** is the phonon scattering matrix.

We write Eq. (S1) in terms of deviational phonon energy density:

$$\frac{\partial g_\mu}{\partial t} + \overrightarrow{\mathbf{v}_\mu} \cdot \nabla g_\mu = Qp_\mu + \sum_\nu \frac{\omega_\mu}{\omega_\nu} W_{\mu\nu}(c_\nu \Delta T - g_\nu) \qquad \text{(S2)}$$

where $g_\mu = \frac{\hbar\omega_n}{Nv}\left[f_\mu - f_\mu^0(T_0)\right]$ and the volumetric heat generation rate Q_μ is replaced by Qp_μ, where Q is the macroscopic volumetric heat generation rate, and p_μ corresponds to how much a given mode is excited by the heating. We assume initial heating is thermally distributed, i.e. $p_\mu = c_u/C$, where $C = \sum_\mu c_\mu$is the heat capacity.

We take the spatial and temporal Fourier transform of Eq. (S2):

$$\widetilde{g_\mu} = \tilde{Q} A_{\mu\nu}^{-1} p_\nu + \Delta\tilde{T}\left(\delta_{\mu\nu} - iA_{\mu\gamma}^{-1} D_{\gamma\nu}\right) c_\nu \qquad \text{(S3)}$$

Where the dummy index indicates summation as in Einstein notation, i.e. $a_\mu b_\mu = \sum_\mu a_\mu b_\mu$, with $D_{\mu\nu} = \delta_{\mu\nu}\left(\Omega + \vec{k} \cdot \overrightarrow{v_\mu}\right)$ and $A_{\mu\nu} = \frac{\omega_\mu}{\omega_\nu} W_{\mu\nu} + iD_{\mu\nu}$. Ω and $\vec{k}$ represent the frequency and wavevector from the Fourier transform.

We sum the Eq. (S3) over all the phonon modes and express the temperature deviation as:

$$\Delta\tilde{T} = \sum_\mu \frac{\widetilde{g_\mu}}{C} \qquad \text{(S4)}$$

We obtain a general expression for temperature response for heating with frequency Ω and wavevector $\vec{k}$ as:

$$\Delta\tilde{T}(\Omega, \vec{k}) = \tilde{Q}(\Omega, \vec{k}) \frac{\mathrm{sum}[\mathbf{A}^{-1}\vec{p}]}{\mathrm{sum}[i\mathbf{A}^{-1}D\vec{c}]} \qquad \text{(S5)}$$

where we define the sum operation of a vector to add up the values of its elements, i.e. $\mathrm{sum}[\mathbf{a}] = \sum_{\mu} a_{\mu}$.

To obtain the time-dependent response of the TTG measurement with a grating period l, we need to substitute the following heating profile:

$$\tilde{Q}(\Omega, \vec{k}) = \delta\left(\frac{2\pi}{l}\right) + \delta(-\frac{2\pi}{l}) \tag{S6}$$

and take the inverse Fourier transform.

3. Ballistic limit

In the ballistic limit, the 1D-PBTE for the TTG takes the from

$$\frac{\partial f}{\partial t} + v_x \frac{\partial f}{\partial x} = 0 \tag{S7}$$

where v_x is the group velocity along the TTG grating period direction, with the initial condition

$$f_o(x) = f_A \cos(qx) \tag{S8}$$

where $q = \frac{2\pi}{l}$, and f_A is the amplitude of the initial distribution due to the laser heating. If the initially excited phonon population is thermally distributed, then $f_A = \frac{C_q Q}{\hbar\omega C}$, where Q is the total heating and C is total heat capacity.

Then the distribution at $t \geq 0$ is:

$$f(x,t) = f_o(x - v_x t) = f_A \cos[q(x - v_x t)] \tag{S9}$$

The measured TTG signal is proportional to the temperature difference between $T(x = 0)$ (the peak) and $T(x = l/2)$ (the null) and can be written as:

$$\begin{aligned} \Delta T &= \frac{< f(0,t)\hbar\omega >}{C} - \frac{< f\left(\frac{l}{2},t\right)\hbar\omega >}{C} \\ &= 2\frac{< f(0,t)\hbar\omega >}{C} \\ &= 2Q\frac{\langle C_q \cos(q v_x t)\rangle}{C^2} \end{aligned} \tag{S10}$$

As in the main context, < > means summation over all the phonon modes.

4. Dispersion of the measured temperature waves and second sound

For a damped temperature wave with wavevector $k = \frac{2\pi}{l}$ and frequency Ω_r and decay rate Ω_i, the temperature excursion can be written as:

$$\Delta T(x,t) = \exp(-\Omega_i t)\cos(\Omega_r t - kx) \quad \text{(S11)}$$

The TTG measured signal ΔT is proportional to the temperature difference between $T(0,t)$ and $T(l/2,t)$:

$$\Delta T(t) = \exp(-\Omega_i t)\cos(\Omega_r t) \quad \text{(S12)}$$

The comparison of Eq. (S12) with the TTG signal is shown in Fig. S7. The Fourier transform of $\Delta T(t)$ is:

$$\Delta T(\omega) = \frac{1}{\Omega_i + i(\omega - \Omega_r)} + \frac{1}{\Omega_i + i(\omega + \Omega_r)} \quad \text{(S13)}$$

This is sharply peaked near Ω_r; near this frequency, we could ignore the second term in Eq. (S13) as its magnitude is much smaller than the first term, and the frequency spectrum can be approximated as:

$$|\widetilde{\Delta T}(\omega)|^2 \approx \frac{1}{\Omega_i^2 + (\omega - \Omega_r)^2} \quad \text{(S14)}$$

Therefore, the frequency of the temperature wave could be obtained by Lorentzian fitting with the temperature response function at a specified wavevector given by Eq. (5) of the main text as shown in Fig. S7.

5. Heat-pulse experiment and three groups of phonons

In the heat-pulse experiment[5,6], two opposite surfaces of the crystal are covered with metal films are deposited onto opposite surfaces of a crystal to serve as the heater and the detector, as shown in Fig.S8a. An electrical pulse is applied to the heater on one side of the crystal. The detector on the right side measures the arrival of the heat pulse via resistance change (Fig. S8a). The propagation speed of the ballistic pulse is close to the sound velocity[5,6]. However, it has been shown theoretically that thermal excitation as conducted in these experiments cannot excite a mechanical wave of significant amplitude[7]. Therefore, the ballistic heat pulses observed in the heat-pulse experiments[5,6] are actually thermally excited ballistic phonons as in three groups of phonons picture. A similar three groups of phonons picture was used to explain the heat-pulse experiments as detailed in Ref. 8. The heat pulses arrived with speeds close to the longitudinal and transverse phonon speeds. The finite sizes of the heater and detector result in a range of propagation directions for phonons that reach the detector, as shown in Fig. S8a, and therefore a range of arrival times is measured yielding an average velocity that is determined from the measurement. For a Debye material of sound velocity **v**, the average speed of ballistic phonons over the solid angle θ that the detector sustained can be estimated as:

$$v_{ave} = \frac{v \sin^2(\theta)}{2[1-\cos(\theta)]} \tag{S15}$$

This average speed is shown in Fig. S8b. For example, as reported in Ref. 9, the detector size was 6 mm, while the sample length was 7.8 mm. Therefore, the maximum solid angle for phonons recorded by the detector could be estimated to be 21°, which yields to a propagation velocity greater than 95% of the sound velocity (Fig. S8b).

6. Contribution from ballistic/diffusive phonons to the TTG signals

The signal in the TTG experiment is proportional to the temperature difference $\Delta T = T(0) - T(l/2)$. The initially excited phonons follow a cosinusoidal spatial distribution and their subsequent transport determines this temperature difference. For a phonon traveling in the positive x-direction, the distance d it travels to reach $\frac{l}{2}$ is $\frac{l}{2} - x$. Whether this phonon's contribution is ballistic or diffusive is determined by the relationship between d and the total MFP Λ_o and R-scattering length Λ_R. If $\Lambda_R < d$, it is diffusive transport. If $\Lambda_o > d$, it is ballistic. Phonons satisfying $\Lambda_o < d < \Lambda_R$ experience hydrodynamic transport. Figure S9a shows in pink and green the ballistic/diffusive components contributions to the measured signal.

The variations of the ballistic and diffusive phonon contributions to the TTG signal with respect to MFP are shown in Figs. S9b and S9c. Summing over all the phonon modes with weights $c_q v_x^2$ as the approximated mode contribution to heat conduction, we obtain Fig. 3a and 3b in the main text. It should be noticed that this is not a rigorous estimation of the ballistic/diffusive contribution, but it should be qualitatively reliable.

7. Mean free path analysis at 200 K

A detailed mean free path analysis is performed to further confirm the observation at 200 K is indeed second sound instead of ballistic signals. Since the ZA phonon dominates the contribution to second sound, we provide a comparison of the grating period and ZA phonon MFP of different scatterings at 200 K (Fig. S10). A characteristic frequency, $k_B T/2\pi\hbar$, is also marked, below which phonons can be readily activated at a given temperature. The average phonon MFP is computed as: $\Lambda_l = \langle C_q v \tau_l \rangle / \langle C_q \rangle$. At 200 K, the Average N-scattering and R-scattering MFP are around 0.2 μm and 5.6 μm respectively. Therefore, our investigated grating periods i.e. 2~3 μm are indeed larger than the MFP of normal scattering and smaller than the MFP of resistive scattering (including Umklapp scattering and isotopic scattering).

8. Thermal zero sound and intrinsic second sound velocity in a Debye material

In this section, we compare the thermal zero sound and intrinsic second sound velocities using the Debye model for phonon dispersion. The TTG signal in the ballistic transport regime is given by Eq. (9) of the main text. For a Debye material with sound velocity v, we can replace the summation over phonon modes in Eq. (9) by integration, and the TTG signal can be written as:

$$\Delta T = \mathrm{sinc}(qvt) \tag{S16}$$

The time of the first dip t_d can be obtained the setting the derivative of the ΔT to zero, which gives:

$$qvt_d \approx 4.4934 \tag{S17}$$

Substitute $q = \frac{2\pi}{l}$ into the above equation, we obtain what we define as the thermal zero sound velocity:

$$v_{tzs} \approx 0.7\, v \tag{S18}$$

Eq. (2a) for a Debye material gives the intrinsic second sound velocity:

$$v_{ss} = \frac{v}{\sqrt{3}}$$

Therefore $v_{tzs} > v_{ss}$ for a Debye material.

9. Characterization of the sample

We observe several graphite crystallites larger than 1 mm in length, which are highlighted in different colors in Fig. S11(a). We performed atomic force microscopy (AFM) to further analyze the surface defects, step edges and grain boundaries. Fig. S11(b) shows AFM height image of the natural graphite crystal. The dashed arrows show step edges where the height changes across the lines. The solid arrows indicate grain boundaries where height does not vary across the line. ImageJ was used to estimate the average area of grains that are surrounded by the step edge or grain boundaries in the AFM image. The average grain area is estimated as 382 ± 270 μm^2 where the largest longer axis could be larger than 100 μm and the typical grain size is larger than 20 μm.

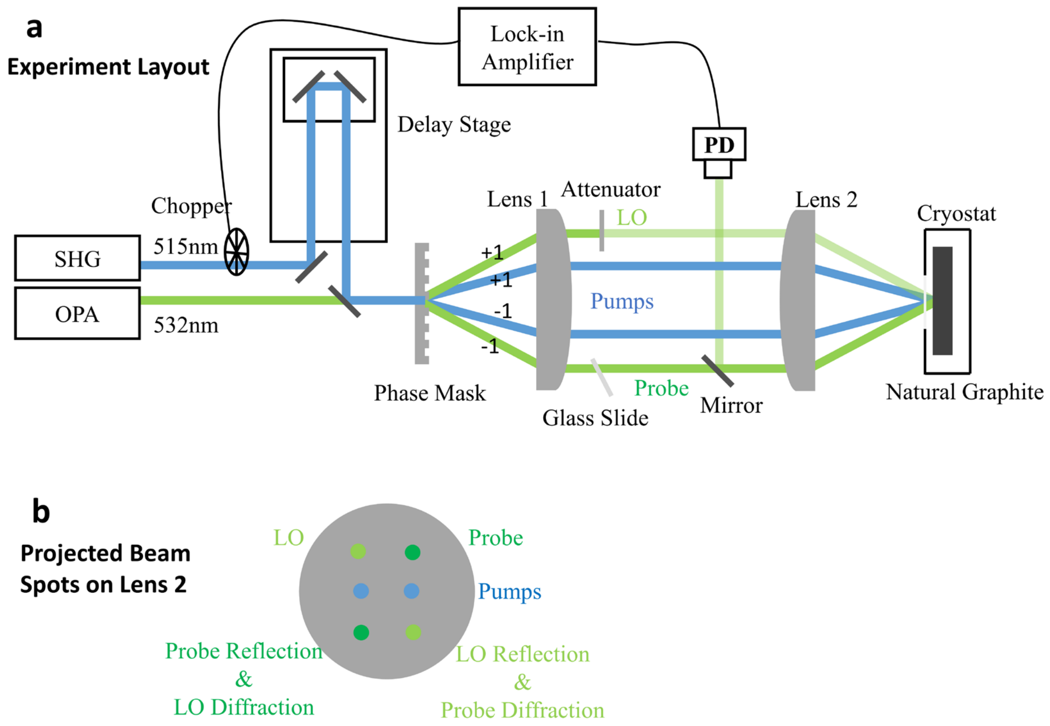

Figure S1. Schematic illustration of the femtosecond laser TTG setup. (a) Top view. The difference in the diffracted angle after the phase mask between the 515 nm and the 532 nm laser beams is exaggerated for better drawing. (b) Side view [from left to right in Fig. S1(a)] of various laser spots at Lens 2.

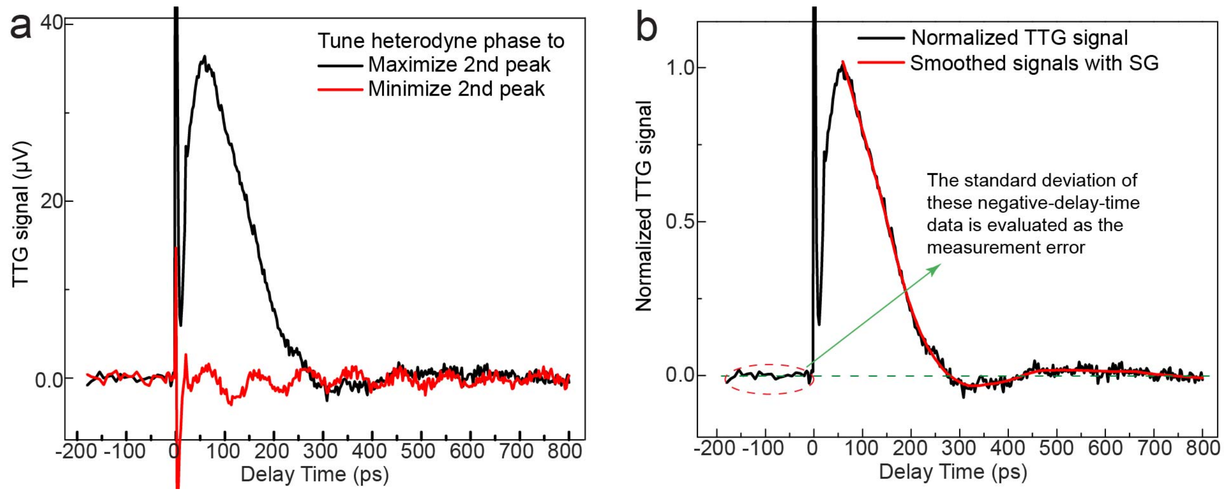

Figure S2. (a) Measured TTG signals at 200 K and 2 μm grating period at two heterodyne phases. One phase is set to maximize the second peak, corresponding to the phase grating signal, while the other phase is set to minimize the second peak, corresponding to the amplitude grating signal. (b) Normalized phase grating signal and the smoothed response obtained using the Savitzky-Golay method. The negative-time-delay signals are used to estimate the measurement error.

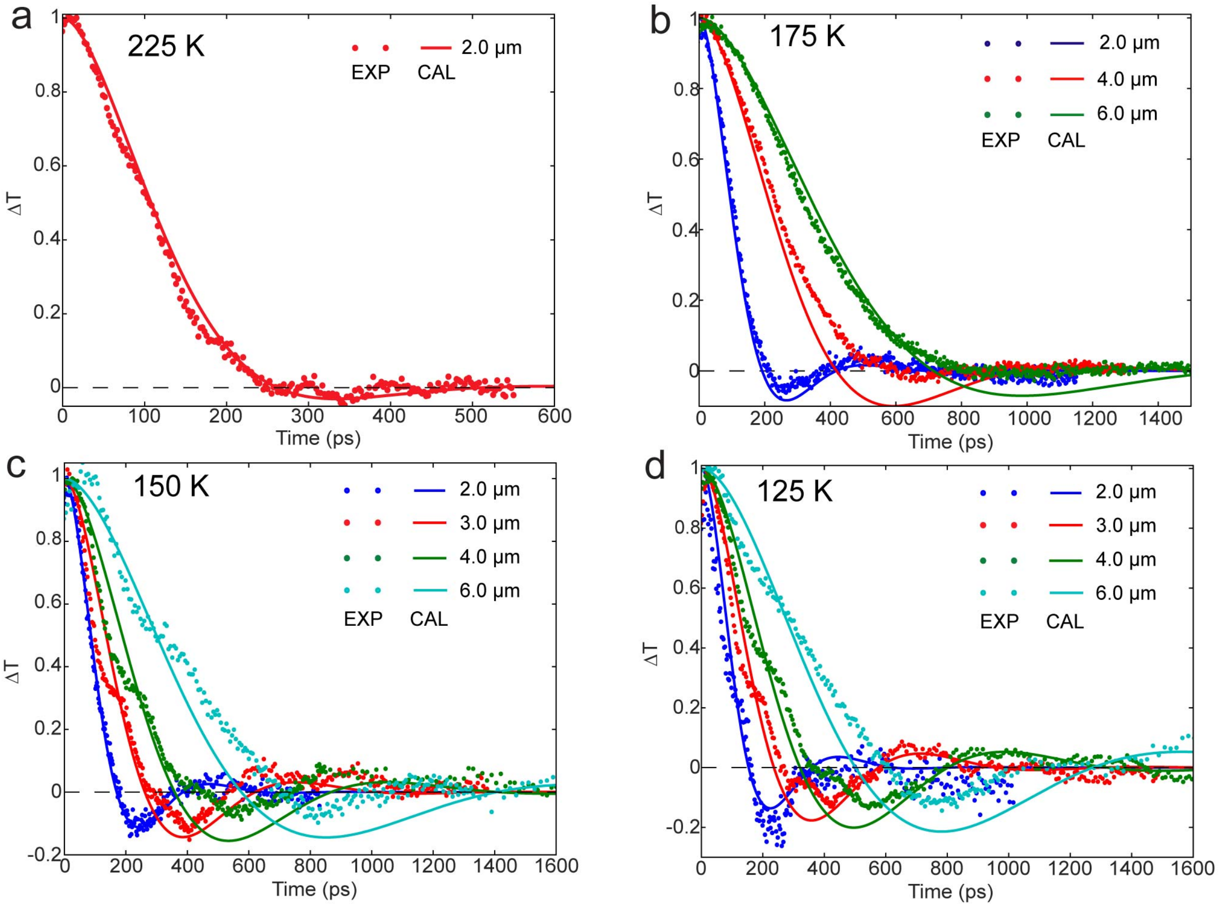

Figure S3. Temperature response of TTG at (a) 225 K, (b) 175 K, (c) 150 K, and (d) 125 K.

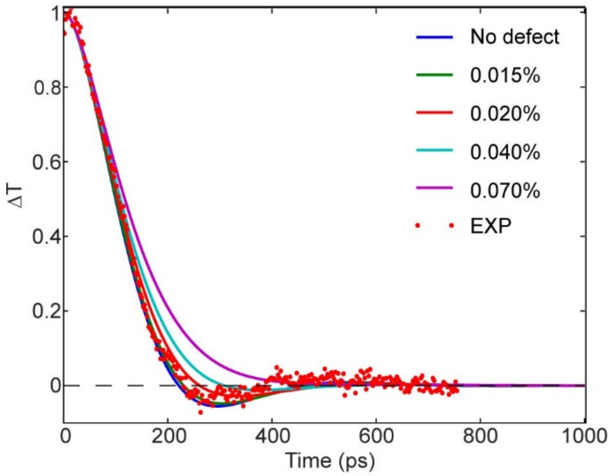

Figure S4. Dependence of the TTG temperature response on vacancy defects at 200 K for a grating period of 2 μm. Dots are the experimental data and curves are the calculations.

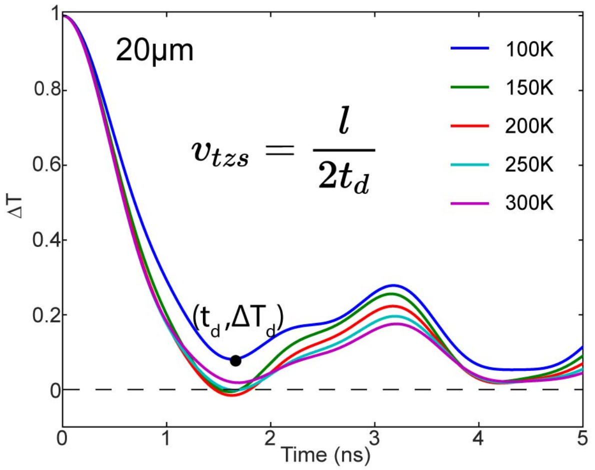

Figure S5. Simulated TTG signal in the ballistic limit at different temperatures.

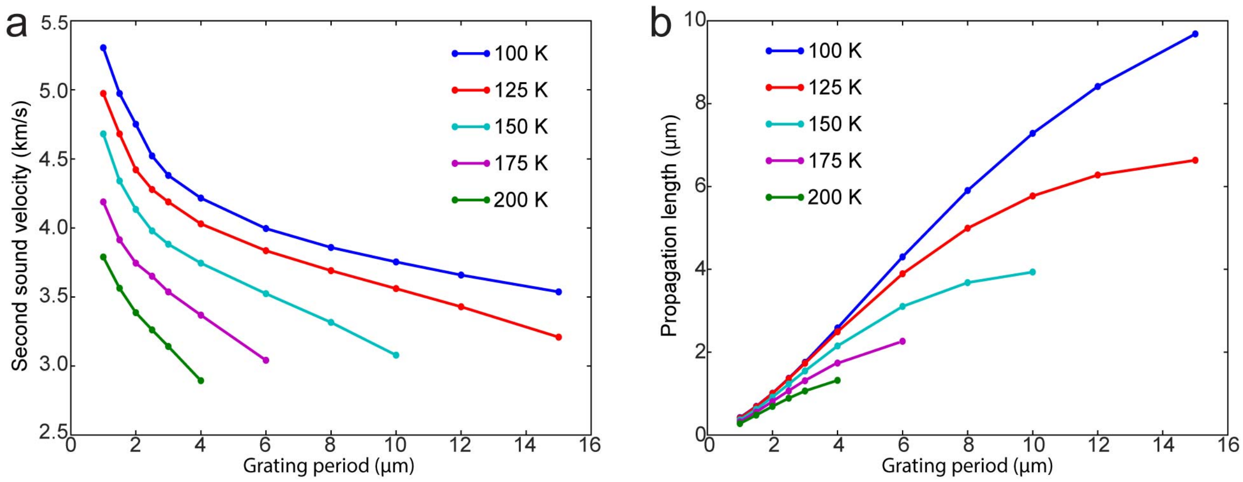

Figure S6 Calculated dependence of (a) second sound speed and (b) propagation length on the grating period at different temperatures.

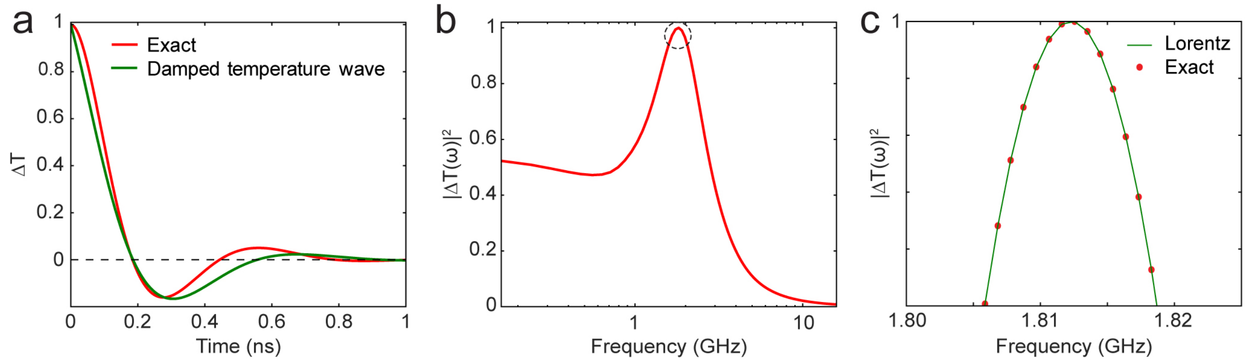

Fig S7 (a) Comparison of a damped temperature wave according to Eq.(S12) with an exact solution of the PBTE (b) The frequency spectrum of temperature response obtained from the Fourier transform of the exact solution, (C) The Lorentz fitting to obtain the real part and imaginary part of the temperature wave.

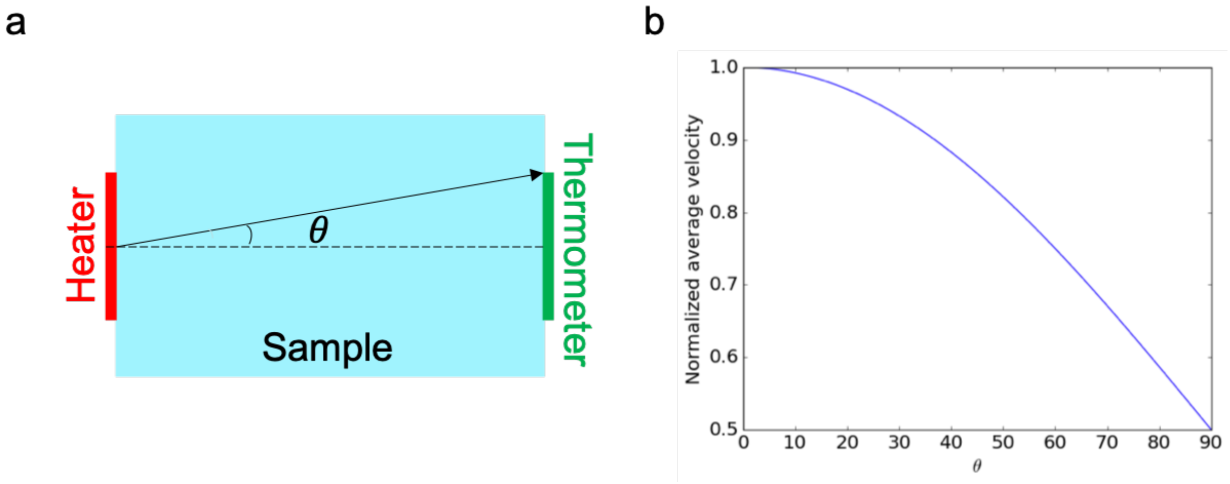

Fig S8 (a) Schematic illustration of the heat-pulse experiment set up, and (b) variation of the average velocity of ballistic phonons with solid angle.

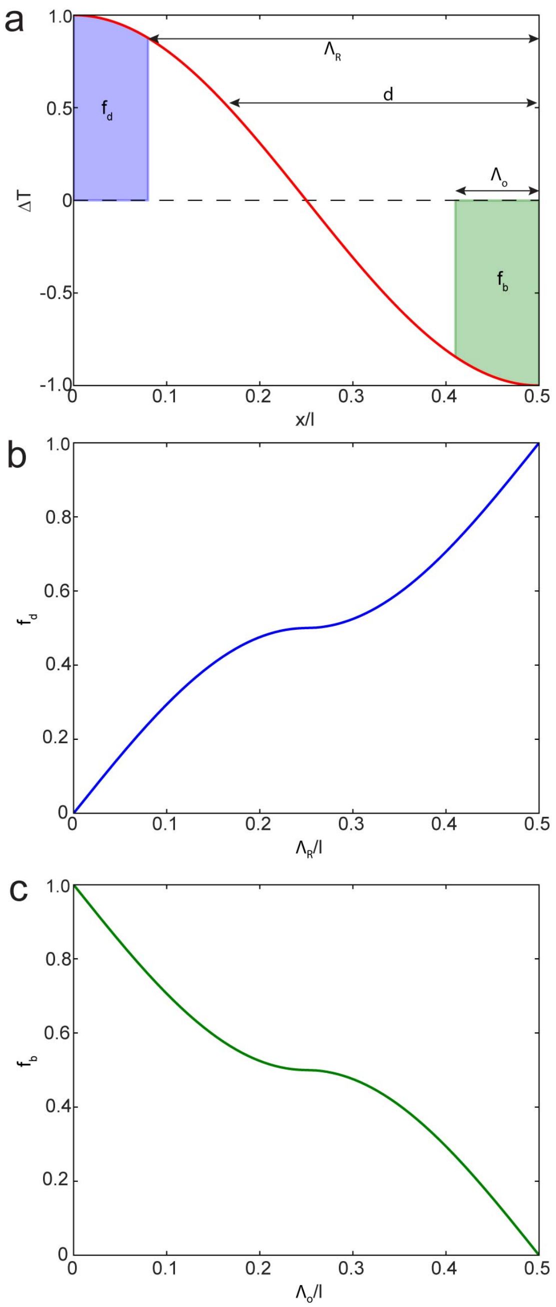

Figure S9. Illustration of diffusive, ballistic, and hydrodynamic phonon contributions to the TTG signal. Λ_o is the total mean free path and Λ_R the resistive mean free path. Phonons are excited between the peak (x/l=0) and the null (x/l=0.5), and their subsequent transport dictates the measured signal. (a) For phonons traveling in the positive x-direction (in reality, excited phonons travel to both positive and negative directions), phonons in the purple region (marked "f_d") reach the temperature valley at x/l=0.5 via diffusive transport, while phonons in the green region (marked "f_b") reach the same location ballistically. Phonons in between the two regions are hydrodynamic contributions. (b) Variation of the diffusive phonon contribution to the TTG signal with respect to R-scattering MFP to grating period l ratio, Λ_R/l, (c) Variation of the ballistic phonon contribution to the TTG signal with respect to total MFP to grating period ratio, Λ_o/l.

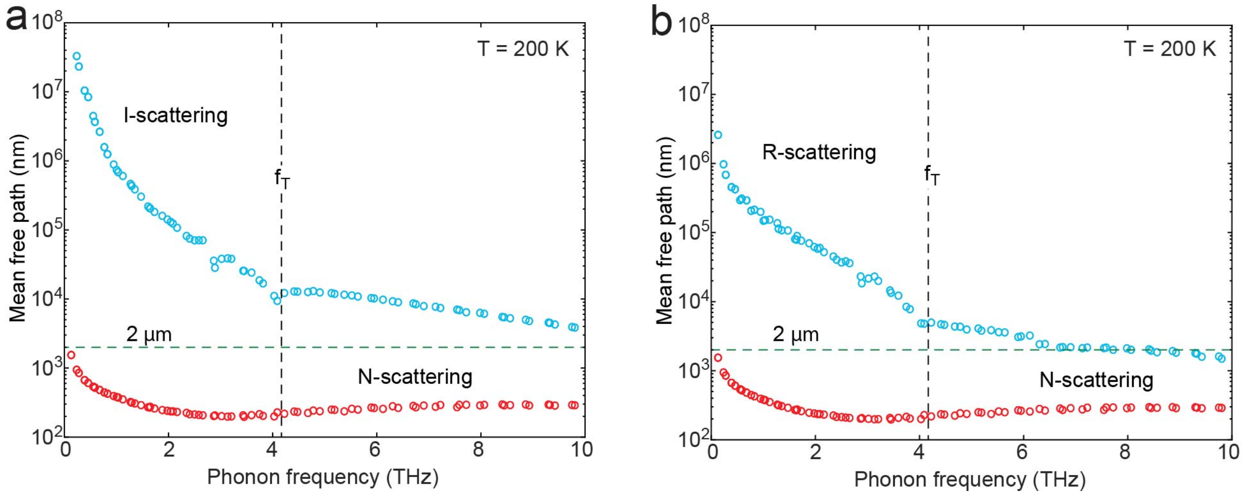

Figure S10. Comparison of mean free path at 200 K (a) N-scattering and I-scattering (b) N-scattering and R-scattering, where the characteristic frequency $f_T = kT/2\pi h$ is marked

a b

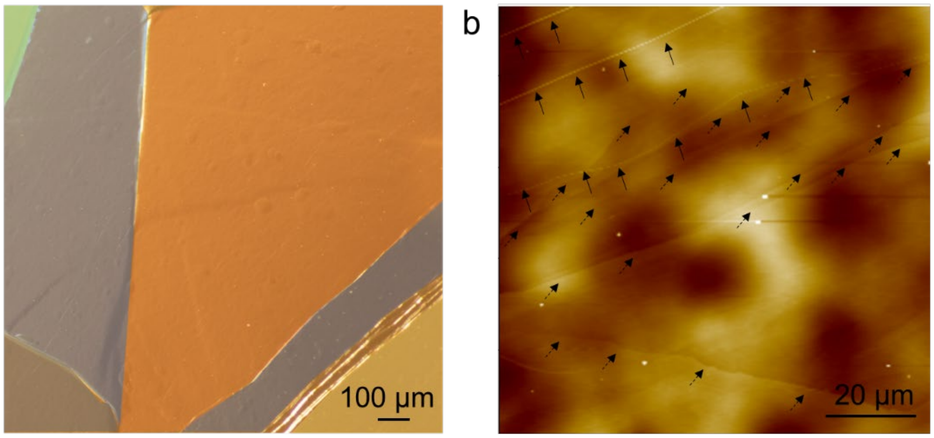

Figure S11 (a) Optical image of the natural graphite crystal. Individual graphite flakes are highlighted in different colors. (b) AFM *z*-height image of the natural graphite crystal. The dashed arrows show step edges where the height changes across the lines. The solid arrows indicate grain boundaries where height does not vary across the line.

References

1. Johnson, J. A. *et al.* Phase-controlled, heterodyne laser-induced transient grating measurements of thermal transport properties in opaque material. *J. Appl. Phys.* **111**, (2012).
2. Huberman, S. *et al.* Observation of second sound in graphite at temperatures above 100 K. *Science* **364**, 375–379 (2019).
3. Savitzky, A. & Golay, M. J. E. Smoothing and differentiation of data by simplified least squares procedures. *Anal. Chem.* **36**, 1627–1639 (1964).
4. Chiloyan, V. *et al.* Micro/nanoscale thermal transport by phonons beyond the relaxation time approximation: Green's function with the full scattering matrix. *arXiv Prepr. arXiv1711.07151* (2017).
5. Narayanamurti, V. & Dynes, R. C. Observation of second sound in bismuth. *Phys. Rev. Lett.* **28**, 1461–1465 (1972).
6. Jackson, H. E., Walker, C. T. & McNelly, T. F. Second sound in NaF. *Phys. Rev. Lett.* **25**, 26–28 (1970).
7. Sham, L. J. Temperature Propagation in Anharmonic Solids. *Phys. Rev.* **163**, 401 (1967).
8. Dreyer, W. & Struchtrup, H. Heat pulse experiments revisited. *Contin. Mech. Thermodyn.* **5**, 3–50 (1993).
9. McNelly, T. F. *et al.* Heat pulses in NaF: onset of second sound. *Phys. Rev. Lett.* **24**, 100 (1970).
