# Supplementary material for: Observation of second sound in graphite over 200 K
Source: Nat Commun. 2022 Jan 12;13:285. doi: 10.1038/s41467-021-27907-z (PMC8755757; doi:10.1038/s41467-021-27907-z)
Supplement: Supplementary file 3 — Lasing Reporting Summary [file 41467_2021_27907_MOESM3_ESM.pdf]

nature research

Corresponding author(s): Keith A. Nelson, Gang Chen

Lasing Reporting Summary

Nature Research wishes to improve the reproducibility of the work that we publish. This form is intended for publication with all accepted papers reporting claims of lasing and provides structure for consistency and transparency in reporting. Some list items might not apply to an individual manuscript, but all fields must be completed for clarity.

For further information on Nature Research policies, including our data availability policy, see Authors & Referees.

Experimental design

Please check: are the following details reported in the manuscript?

1. Threshold

Plots of device output power versus pump power over a wide range of values indicating a clear threshold

☐ Yes
☒ No

The laser is working at a fixed pump power level (10W), which is the factory setting. By monitoring the lasing intensity and repetition rate, a selfcheck program is running to confirm the stability of mode-locked lasing at the output.

2. Linewidth narrowing

Plots of spectral power density for the emission at pump powers below, around, and above the lasing threshold, indicating a clear linewidth narrowing at threshold

☐ Yes
☒ No

The laser used is femtosecond pulse. There is no need for linewidth narrowing.

Resolution of the spectrometer used to make spectral measurements

☐ Yes
☒ No

No wavelength-dependent experiment is reported in the manuscript.

3. Coherent emission

Measurements of the coherence and/or polarization of the emission

☐ Yes
☒ No

No coherence-related or polarization-dependent experiments are reported in the manuscript.

4. Beam spatial profile

Image and/or measurement of the spatial shape and profile of the emission, showing a well-defined beam above threshold

☒ Yes
☐ No

The " Methods" section.

5. Operating conditions

Description of the laser and pumping conditions
Continuous-wave, pulsed, temperature of operation

☒ Yes
☐ No

The "Methods" section and Figure S1

Threshold values provided as density values (e.g. W cm^{-2} or J cm^{-2}) taking into account the area of the device

☒ Yes
☐ No

The "Methods" section.

6. Alternative explanations

Reasoning as to why alternative explanations have been ruled out as responsible for the emission characteristics
e.g. amplified spontaneous, directional scattering; modification of fluorescence spectrum by the cavity

☐ Yes
☒ No

The important emission characteristics of the laser in this manuscript are the pulse width, the wavelength, and the pump fluence, which have been measured and stated in the "Methods" section.

7. Theoretical analysis

Theoretical analysis that ensures that the experimental values measured are realistic and reasonable
e.g. laser threshold, linewidth, cavity gain-loss, efficiency

☐ Yes
☒ No

The measured characteristics, such as pulse width, wavlength, laser power, are all within the specification range of the laser equipments.

8. Statistics

Number of devices fabricated and tested

☒ Yes
☐ No

The "Methods" section and Figure S1

Statistical analysis of the device performance and lifetime (time to failure)

☐ Yes
☒ No

The devices used in the manuscript, such as lock-in amplifier, delay stage, photo detector, optical chopper, have sufficient long lifetime (tens of years).

March 2018
